# COVID-19 in forensic psychiatry settings: The unique vulnerability of patients in secure services

**DOI:** 10.1192/j.eurpsy.2021.1004

**Published:** 2021-08-13

**Authors:** N. Basrak, N. Mulcrone, S. Sharifuddin, Z. Ghumman, N. Bechan, E. Mohamed, M. Murray, H. Rajendran, S. Gunnigle, M. Nolan, T. Quane, M. Terao, T. Hoare, K. Kirrane, H. Kennedy, M. Davoren

**Affiliations:** 1 National Forensic Mental Health Service, Central Mental Hospital Dundrum, Dundrum, Ireland; 2 The Dundrum Centre For Forensic Excellence, Trinity College Dublin, Dublin, Ireland

**Keywords:** COVID-19, forensic psychiatry

## Abstract

**Introduction:**

Secure forensic mental health services treat patient with high rates of treatment resistant psychoses, typically schizophrenia. These groups have high rates of obesity and medical co-morbidities. Population based studies have identified high risk groups in the event of SARS-CoV-2 infection, including those with long term medical conditions.

**Objectives:**

The aim of this study was to compare the vulnerability to serious adverse outcome in the event of COVID-19 infection in a forensic psychiatric patient population.

**Methods:**

All patients of a complete National Forensic Mental Health Service (n=141) were rated for risk of adverse outcome in the event of SARS-CoV-2 infection, using two structured tools, the COVID-AGE tool and the COVID-Risk tool.

**Results:**

Eighty-two patients (58.2%) met criteria for obesity, 32 had type II diabetes and 28 were hypertensive. Mean chronological age was 45.5 years (SD 11.4, median 44.1), while mean COVID-AGE was 59.1 years (SD 19.4, median 58.0), mean difference 13.6 years (SD 15.6) paired t=10.9, df=140, p=0.000. Three patients (2.1%) were chronologically over 70 years compared to 40 (28.4%) with a COVID-AGE over 70 (X2=6.99, df=1, p=0.008, Fishers exact test p=0.027).

**Conclusions:**

These risk assessments may identify the extent of increased risk among a uniquely medically vulnerable patient group. Patients in secure forensic psychiatric services represent a high-risk group for adverse outcomes in the event of SARS-COV-2 infection. Population based cocooning and self-isolating guidance based on chronological age may not be sufficient. There is an urgent need for better physical health research and treatment in this group.

